# 3D-Printing Physical Activity in Youth: An Autotopographical Approach to Behaviour Change

**DOI:** 10.3390/ijerph20021530

**Published:** 2023-01-14

**Authors:** Melitta A. McNarry, Rachel L. Knight, Sam G. M. Crossley, Paula Foscarini-Craggs, Zoe R. Knowles, Parisa Eslambolchilar, Kelly A. Mackintosh

**Affiliations:** 1 Applied Sports, Technology, Exercise and Medicine (A-STEM) Research Centre, Department of Sport and Exercise Sciences, A104 Engineering East, Faculty of Science and Engineering, Swansea University, Bay Campus, Swansea SA1 8EN, UK; 2 Centre for Trials Research, Cardiff University, Cardiff CF10 3AT, UK; 3 Physical Activity Exchange, School of Sport and Exercise Sciences, Liverpool John Moores University, Liverpool L3 5UX, UK; 4 School of Computer Science and Informatics, Cardiff University, Cardiff CF10 3AT, UK

**Keywords:** sedentary behaviour, children, adolescents, qualitative, focus groups, autotopography, self-reflection, peer-comparison

## Abstract

The conceptualisation and visualisation of physical activity through 3D-printed objects offers a unique means by which to elicit positive behaviour change. This study aimed to explore whether 3D-printed models of physical activity obtain autotopographical meaning in youths and the influence of such models on their sense of personal and social identity. Following participation in a seven-week faded intervention, whereby habitual physical activity was measured and used to create individual 3D models, the views of 61 participants (36 boys; 10.9 ± 3.0 years) were explored via semi-structured focus groups. Within the over-arching theme of ‘3D-Printed Models’, key emergent sub-themes were structured around ‘Autotopography’, ‘Reflection’, ‘In-group norms’, and ‘Significant others’. Investing meaning in the material representations facilitated social activation and self-reflection on their own behaviour, both of which are key elements of identity formation. The influential role of significant others (familial and peers) within initial model interpretation and their potential long-term efficacy as a behaviour change approach was highlighted. 3D-printed models present a novel concept and intervention approach and may represent a useful component within behaviour change engagement strategies in children and adolescents.

## 1. Introduction

The deleterious physiological and psychosocial impacts of physical inactivity are well-documented; for a decade, physical inactivity has been recognised as the fourth leading cause of mortality globally [1]. The World Health Organization (WHO) have set a global target of decreasing physical inactivity across the population by 10% by 2025 and 15% by 2030 [2]. Despite the importance of children (aged 5–18 years) achieving at least 60 min of moderate-to-vigorous physical activity (MVPA) every day, more than 80% of adolescents globally do not currently meet this guideline [3]. However, interventions developed using social cognitive models focusing on barriers and facilitators to physical activity, as well as self-regulation to sustainably increase youth’s physical activity (PA) levels, have had limited success [4], and radical changes to current intervention approaches to increasing PA are seemingly required [5].

Recent technological advances offer numerous opportunities to tackle physical inactivity, with one such novel approach focusing on the conceptualisation and visualisation of PA through 3D-printed objects. Specifically, 3D-printed objects enable the transformation of a performance (e.g., habitual PA) into a material object [6]. Indeed, research with adults supports the potential efficacy of 3D representations of heart rate and PA to enhance participant motivation [7,8,9], with more recent work suggesting that 3D models of PA may increase children and adolescents’ understanding of, and motivation to engage in, PA [10,11,12]. Increased understanding of PA levels through visualisation aligns with the principle of objectification, which occurs when individuals take an unknown concept and make it concrete [13,14,15]. However, whilst promising, studies to date have focused on the short-term ability (e.g., 3–7 weeks) of 3D-printed objects to raise an individual’s self-reflection, awareness, and motivation for PA, with no long-term follow-up [7,9]. If such interventions are to be successful at sustainably increasing PA levels and precluding the novelty effect as a predominant explanation of their success, they must elicit long-term behaviour change.

A key element of sustainable behaviour change is that of an individual’s personal and social identity [16,17,18], with a growing body of research supporting the efficacy of manipulating an individual’s, and thus group’s, social identity to elicit collective behaviour change at a societal level [19,20,21]. Such studies utilise the concept of an “in-group norm” which is viewed as being internally, rather than externally, driven. This concept revolves around the notion that when individuals categorise themselves as similar to others, they internalize the characteristics they have in common, and define the group as being distinct from comparison “outgroups” [20,22,23]. One potential characteristic that could be adopted to create such an “ingroup norm” would be a common level of PA reflected in a 3D-printed object. Indeed, using a 3D-printed object to represent PA data is more publicly visible to peers compared to data collected and displayed on a private computer or smartphone [6]. For these reasons, the increased visibility of PA levels through a 3D-printed object may support two forms of social incentive, competition and co-operation, where youth can compare their PA levels against others, or motivate each other to achieve a common PA goal, respectively [7]; both of which may strengthen an individual’s social identity [24].

Supporting the potential role of 3D-printed models in eliciting positive behaviour change, it is argued that ubiquitous tangible technologies that allow individuals to annotate and provide narrative explanations for their experiences are required [25]. It has previously been reported that when individuals amassed and displayed collections of objects which reflected their journey of self-improvement, these objects gained autotopographical meaning and importance according to the time and effort that was involved in their creation [7,25]. Autotopography is a behaviour in which material objects are used to construct a sense of identity by cultivating the physical environment [26], thereby acting as a physical map of memory, history, and belief [25]. For example, Stusak et al. [9] found that adults readily displayed their 3D-printed models of PA in their workspace (on top of computer monitors), whilst Crossley et al. [10] observed this in children in their home environments (mounted to their bedroom walls). This way, the 3D-printed models allowed individuals to display their PA achievements in prominent places, to be seen, reflected upon, and shared with significant others via conversations [25]. For youth, the applicability of these findings to autotopographical ideologies and constructs is presently unclear, with little known regarding the effect of 3D-printed representations of PA on their personal and social identity. The purpose of the present study was to explore whether 3D-printed objects representing PA levels obtain autotopographical meaning in youths, as well as the influence of these models on their sense of personal and social identity.

## 2. Methods

**Figure 1:**
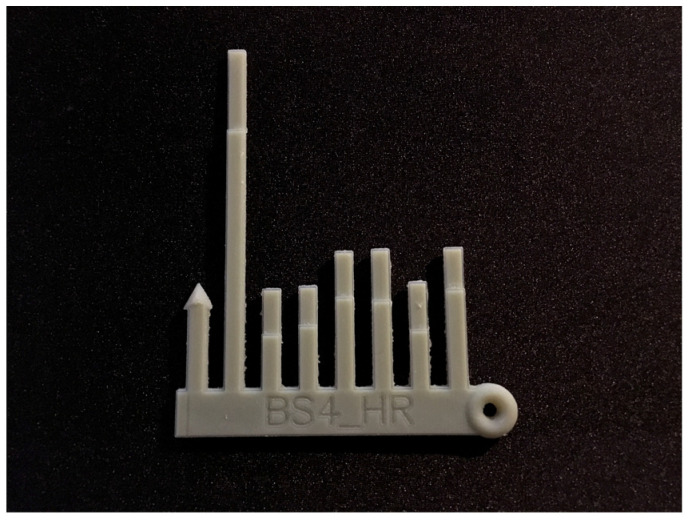
3D-printed model of moderate-to-vigorous physical activity across a week.

The current study is part of a research programme investigating the efficacy of 3D-printed models of PA as an educational and motivational tool in children and adolescents [10]. Briefly, this programme consisted of a seven-week faded intervention, whereby feedback frequency decreased over time. Participant’s seven-day habitual PA was measured via hip-worn accelerometers (wGT3X+-BT; ActiGraph LLC, Pensacola, FL, USA) and used to generate personal 3D-printed models previously co-developed with participants (Figure 1) [12] to represent MVPA achieved each day across a week, compared to the current international PA guidelines (at least 60 min MVPA). PA was represented by bars in the model, and more PA resulted in larger lines.

In total, 61 participants (36 boys; 10.9 ± 3.0 years) recruited from the wider intervention study, as described in detail elsewhere [10], provided informed parental/carer consent and child assent to participate in this study. Specifically, thirty-one children (7.9 ± 0.3 years; 17 boys) and thirty adolescents (13.8 ± 0.4 years; 22 boys) were included. A purposive sampling approach was employed, whereby only participants who took part in the overall intervention and received 3D models, were approached. Following prior stratification into high and low socioeconomic status groups, based on the percentage of pupils eligible for free school meals, 34 and 27 individuals, respectively, were invited to take part in focus groups. Focus groups were employed as they enable a researcher to tap into youths’ collaborative experiences through the encouragement of group dialogue and the exchange of ideas [27], empowering youth as the experts [28]. All procedures were approved by the Swansea University Ethics Committee and were conducted in accordance with the Declaration of Helsinki (reference: PG/2014/40).

### 2.1. Procedures

On intervention completion, participants were asked to participate in a semi-structured focus group conducted by SGMC at school during school hours in an unbiased and non-directive manner [29]. Using a semi-structured approach which encouraged participants to articulate in detail and from their own viewpoint, participants were asked about their thoughts, personal interactions, and interactions with family and peers regarding their personalised 3D models. The questions were open-ended, and probes were used to gain further insight where necessary. Example questions included the following: “In what way/s did you compare your models to others?”; How did you feel when you compared your 3D models with friends/classmates?”; “Why did you/didn’t you compare your models?”; “What did you/your family/other adults say about your models?”. The 20 pre-determined questions were reviewed and discussed by SGMC, MAM, PE, and KAM, and further verified by a Health and Care Professions Council Registered Psychologist. The full interview schedule is provided in the online Supplementary Materials.

Focus group discussions (five primary school, six secondary school) typically involved 6–7 participants to allow for lively, yet manageable, interactions [29,30], with the exception of two groups, which had two and three children, respectively. Whilst these focus groups were shorter (29.8 ± 4.9 min compared to 45.2 ± 6.4 min), the depth and opportunity for interaction were comparable to those obtained in the larger focus groups. Both single and mixed-sex focus groups were conducted [31]. All focus group sessions were completed within the school environment and during the school day, either within a familiar classroom, or the school library, to provide comfort, and reduce anxiety [32]. Participants were seated in a circular arrangement around a table to create a relaxed and informal atmosphere [29], maximizing social interaction and observer involvement [33]. To ensure each of the group members was comfortable with talking aloud, and to create an environment in which sharing and listening were valued, an ice breaker question was used [34]. All focus groups were recorded using a digital (Olympus DM-520 digital voice recorder, Shinjuku, Japan) and video (Sony Handycam HDR-PJ540, Minato, Japan) recorder and were transcribed verbatim. In total, 11 focus groups lasting 42.4 ± 8.6 min were conducted, resulting in 260 pages of Arial size 12 double-spaced raw transcription data.

### 2.2. Data Analysis

All transcripts were initially read in an active way by PF-C, searching and noting for meanings and patterns within the dataset [35]. Following the initial data immersion process, transcripts were then thematically analysed both deductively and inductively using data coding and identification of themes by a manual cut and paste technique [35]. Transcripts were first deductively analysed using the semi-structured interview questions and the social identity and autotopography constructs, as a thematic framework. Additionally, an inductive approach was taken to code and identify any new emergent themes to aid the exploratory nature of the research [36]. Analyses for any additional themes were not limited to evaluating responses to individual questions but purposely took into account the entire transcript. This deductive and inductive approach allowed for all the data to be organised into meaningful groups that were considered pertinent to the research question. All codes were then sorted into potential themes by collating all the relevant coded data extracts within the newly identified theme. Frequency counts were calculated for main- and sub-themes with indicative verbal statements chosen by PF-C and then represented diagrammatically using a pen profile approach [12,37]. Pen profiles provide a visual representation of key emergent themes and the frequency with which they occur, thereby giving an indication as to the weighting of each theme within the current sample population [37,38]. To ensure trustworthiness of the analyses, reverse triangulation was conducted whereby members of the research team (ZRK and KAM) challenged the key themes and their interpretation. Themes that did not have enough supportive data or were too diverse for the research question were discarded by PF-C. Finally, the pen profiles were critically reviewed by all authors, allowing further interpretations of the data until a final consensus was reached.

## 3. Results

**Figure 2:**
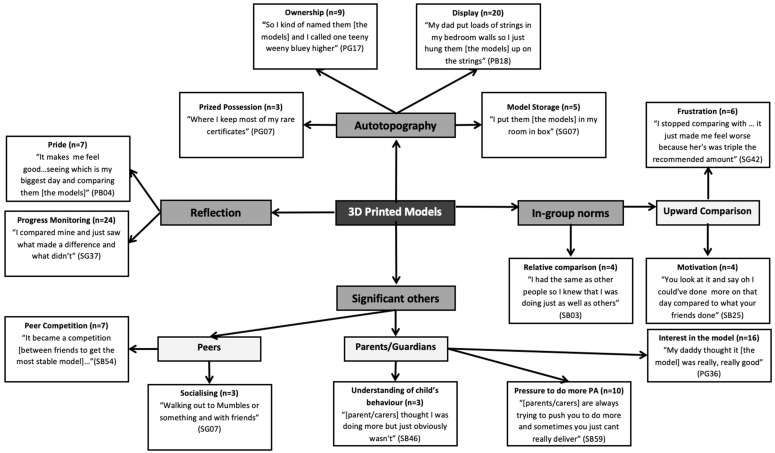
Pen profile representing youth’s interactions with the 3D models.

A pen profile was constructed from the analysis outcomes to represent youth’s interactions with the 3D models (Figure 2). Consistent themes were identified irrespective of age, sex, or level of engagement; data were therefore pooled for final analysis. However, there were some differences in the manifestation of themes across some of these parameters that are outlined accordingly. Unique identification codes were used to ensure anonymity of participants within all transcripts: P (primary), S (secondary), B (boy) or G (girl), followed by participant number. The overall theme ‘3D-Printed Models’ examined the way the models were utilised as an extension of participant’s personal and social identity. As shown in Figure 2, within this overarching theme, the key emergent themes identified from the interview data were structured around ‘Autotopography’, ‘Reflection’, ‘In-group norms’ and ‘Significant others’. Additional linking sub-themes represent frequency counts and verbatim quotes.

### 3.1. Autotopography

The 3D model of PA as an object of *autotopography* manifested itself in five different ways. Youth (*n* = 20; 32.8%) most commonly cited how they would *display* their 3D model “*on my desk*” (SG38) or “*on my shelf*” (SB19), as having the model “*on visual display [would] kind of motivate you to do more [PA]*” (SB29). Participants discussed how they would *store their models* (*n* = 13; 21.1%). Some of the youth would carry their models with them (*n* = 5; 8.2%)*,* in their school “*bag on the keychain…with my keys*” (SB16) or inside their “…*pencil case so I will get them [the 3D models] out and have a look at them*…” (SG15). Others (*n* = 5; 8.2%) described how they would leave their models at home, “*in my room in a box*” (SG07) or in a “*safe drawer*” (PG09). Interestingly, youth who stored their models of PA reported how they would “…*take them [the models] out and look at them*” (SG44). A smaller number of participants (*n* = 3; 4.9%) kept their models with *prized possessions,* such as their “*diary because I’ve got a key for my diary*” (PG13) or with other significant objects, such as their “*rare certificates*” (PG07).

There were sex differences in the way that the theme of autotopography manifested itself. Boys in the study were more likely to discuss displaying their model (*n* = 15/20; 75%), whereas girls were more likely to mention how they stored their models after they received them (*n* = 10/13; 76.9%). There was also an age-based difference, with only the secondary school-aged participants discussing carrying their models with them, the primary school-aged participants were more likely to discuss storing their models at home. Finally, the 3D models gained autotopographical meaning through youth’s discussions regarding the sense of *ownership* they had of the model and their PA (*n* = 9; 14.8%) because “*it [the model] is yours and you’ve done it [the PA]*” (SB16) and “*it kind of like makes you work harder, like then you know what you’re getting out of it like you know that you’re going to get like model*” (SB25).

### 3.2. Reflection

The 3D models prompted most of the participants to *reflect* on their PA levels (*n* = 31; 50.8%). Irrespective of age, more boys (n = 14/24; 58.3%) reported comparing their own 3D models across time as a form of *progress monitoring* compared to girls (*n* = 10/24; 41.7%). This was also more common in the secondary school-aged participants (*n* = 18/24; 75%) compared to primary school-aged participants (*n* = 6/24, 25%). In contrast, primary school students (*n* = 6/7; 85.7%) were more likely to express a sense of *pride* regarding their model, making them “*feel good*” (PB20) and “*proud*” (SB16) of what their levels of PA: “you, *just like, look how much you’ve...like ran and walked and you are just proud*” (PG7).

### 3.3. In-Group Norms

In comparison to adolescents (*n* = 10/14; 71.4%), very few primary school children (*n* = 4/14; 28.6%) compared their models and by extension their PA with their peers. Participants (*n* = 10, 16%) most commonly demonstrated an *upward comparison* (i.e., comparing their models with those children who did more PA) through either *frustration* (*n* = 6; 9.8%) because “*I felt sad, because mine [3D model] wasn’t bigger than his [peers]*” (PM21) or a *motivation* (*n* = 4; 6.6%) to do “*better than everybody else*” (SM25). A smaller proportion of participants (*n* = 4; 6.6%) mentioned how their model was *relative* to their peers’ models or “*around average*” (SM02) and that “*comparing myself to other people in classes, they [peers] haven’t really done as much [PA], I thought that they [peers] would be more active*” (SM21). At times, the comparison resulted in negative emotions, like embarrassment, but also a reflection on the social perception of what the model represented in relation to a person’s identity (…*I forgot to wear it and they don’t believe you they just think you are really lazy, and it makes you feel a bit annoyed at how, what you should have done last week* (SM25)).

### 3.4. Significant Others

Within the higher order theme ‘*Parents/Guardians’*, youth (*n* = 3; 4.9%) mentioned how the 3D model changed their parents/guardians understanding of their PA behaviour, as “*my parents didn’t think I already did much exercise*” (SB51) and they it “*sort of like surprised [them]…what the average [amount of PA] was*” (SB53). In some cases, parent/guardian’s increased *understanding of their child’s behaviour* from the models resulted in some youth (*n* = 10; 16.4%) expressing feeling *pressure to be more physically active*, “*to get out the house and do activities*” (SB60) and “*to start doing more sports*” (SB25). Boys were more likely feel pressure to do more exercise (*n* = 8/10; 80%). Participants (*n* = 16; 26.2%) also described how their parents/guardians were interested in “*looking at the models*” (SG06) and intrigued as to “*what did you do on that day*” (SG44).

Within the higher order theme ‘*Peers*’, youth (*n* = 7; 11.4%) expressed that “*obviously it’s quite competitive in school so you want yours [3D model] to be the biggest*” (SB16) because you get “*bragging rights*” (SB03), although this *peer competition* was only evident in boys in the current study. Moreover, some youth (*n* = 3; 4.9%) used the creation of 3D models of PA as an opportunity to *socialise* with friends through engagement in further PA “*to try and meet the [3D model target], like going out on bike rides and going out with friends*” (SB54).

## 4. Discussion

Previous research has established that tangible representations of PA can influence both adult’s [7,9,25] and youth’s [10,11,12] understanding of their PA levels, and motivation to be more active. Indeed, PA identity, through the process of creation and being displayed for others to see, served as a means of connecting with key social groups [7,9,10,11,12,25]. Building on research to date, the aim of the current study was to explore the autotopographical meaning associated with 3D-printed, tangible representations of personal PA levels and to ascertain their influence on youth’s personal and social identity. It was identified that youth invested meaning in material representations of PA and that this autotopographic behaviour subsequently, to some degree, influenced their sense of personal and social identity. Furthermore, by facilitating social comparisons between peers, the 3D-printed models of PA influenced participant’s perceptions and social categorisation of others.

Physical activity is well-evidenced to be protective against numerous primary and secondary causes of morbidity and mortality, including, but not limited to, cardiovascular disease, type II diabetes and some cancers [39]. In addition to the direct benefits associated with PA, it is also crucial in the development, maintenance, and improvement of self-identity [40]. However, despite recognition of this and the consequent need to find effective strategies for PA promotion, the majority of approaches have, to date, been focused on social cognitive models. Such approaches orientated towards the removal of barriers to action, enhancement of the perceived positive relative to negative consequences of PA, and the development of skills to regulate behaviour [40], demonstrate only modest success in behaviour prediction and change [4]. 3D-printed models offer a novel approach to PA promotion and behaviour change; these models may challenge youth’s identities and established schema, providing a stimulus to promote change.

Identity and schema are two very similar, or even commensurate [41], constructs which have been reported to be amongst the strongest known correlates of PA behaviour [40]. Specifically, identities are considered components of a multi-dimensional self-concept, hierarchically organised according to how one views oneself in a given role [42]. Particularly relevant to the concept of 3D printing, these standards act as comparators to actual behaviour and are activated in situations, such as when receiving a tangible model, where identities are either aligned or mismatched to one’s behaviours [40]. While identities are focused on personal and social standards and the motivation to match these standards to behaviour, schema are integral to the processing and initiation of behaviours through the efficient screening of relevant information [43].

In this study, it is likely that the repeated provision of 3D models of PA was influential in the youth’s development of a PA-related schema, and that identities were informed and adapted based on the information they provided relative to previous weeks and their peers. Indeed, future research should integrate measures of identity/schema (through questionnaires or computer-based word matching tasks, i.e., [44] to ascertain the role of behaviour accordance versus discordance in the efficacy of 3D-printed models to sustainably promote PA in youth. Given the potential link between identity and motivation via broader social, cultural, and personal contexts than typically considered in popular models of behaviour change [45], such research may advance our understanding of successfully and sustainably eliciting positive behaviour change in children and adolescents.

Social activation through social comparisons are suggested to be a key element to identities, with identities partly formed from a comparison to others and feelings of belonging [40]. Whilst research suggests that not all individuals are pre-disposed to use social comparison methods [46,47], this appeared to be predominantly age-related in the current study. Specifically, secondary school-aged youth frequently engaged in comparisons, whilst primary school-aged children were more likely to comment on how their 3D model of PA made them feel, i.e., proud. It is postulated that the primary school age group saw their models as an opportunity to build their PA skills (e.g., more football, gymnastics, climbing, and skateboarding) and gain the confidence to start new activities (e.g., “*I’m in dance class now*” PG11 and “*I started playing football*” PB02) and that they did this by comparing their own models across time [48]. In contrast, the 3D-printed models of PA afforded secondary school-aged youth with opportunities to develop social relationships through competition with peers, and socializing with friends during exercise and PA. Indeed, it has previously been reported that having peers to share their active time with is important, particularly for adolescent girls [16], when self-awareness and pre-occupation with self-image dramatically increase [49].

Interestingly, youths also used their models of PA as a proxy for adherence to perceived social norms. These social comparisons may guide how individuals identify, interpret, and label internal states, especially when these states are novel experiences such as those elicited during attempts to alter health behaviours [50]. The current study explored the extent that 3D models of PA were used in social comparisons and provides credible foundations on which this could be initiated, as well as demonstrating methods appropriate to understand participant experience. Nonetheless, future research should further explore how the 3D-printed models facilitate social comparisons and what impact this has on long-term behaviour change. Whether through the discussions surrounding social comparison, or the competition that evolved from receiving the models, youths reported how the 3D-printed models of PA represented their hard work and thereby gained autotopographical meaning, (as per prior research) [7,25] because of the time and effort involved in their creation.. The models therefore have the potential to be considered as a public representation of self; a key element of the 3D models that distinguishes them from more conventional digital feedback.

The tangible nature of 3D models, however, not only makes them more public and visible, but also allows them to be touched, explored, carried, and compared with others [51]. Indeed, some of the youths described how the models evoked a strong sense of ownership. This factor alone may provoke youth to take personal responsibility for their health and wellbeing by engaging in sufficient PA levels. Further research is required to explore how the children’s shifting identities in relation to PA changes their interactions with, and feelings towards, their personal models.

It is pertinent to note certain potential negative consequences of the models gaining autotopographical meaning, including that participants may not want to display or interact with the models if they convey information that can negatively influence their social identity, as previously noted by Goodyear et al. [52]. Some youth in the current study also reported being concerned about forgetting to wear the activity monitor and the potential negative perception (i.e., being lazy) conveyed to peers because the model did not reflect their true PA: “…*when I show this to people they are all like ‘you haven’t done much’ and I was like ‘yeah, I forgot to wear it’ and they don’t believe you… just think you are really lazy and it makes you feel a bit annoyed*” (SB25).

According to the theory of co-experience, the experiences an individual has and the interpretations that are made of them are influenced by the physical and/or virtual presence of others [53]. This potential limitation is reflected in previous studies [54,55] and is a commonly cited limitation to the use of living metaphors and avatars for personalised PA and health promotion [56,57]. Lin et al. [57] developed an online living metaphor visualisation through an animated fish whose emotional state and size changed to happier and larger, respectively, in response to increased levels of PA, and vice versa. The authors reported that those individuals who were inactive disengaged with the online platform as a consequence of their fish looking unhappy in comparison to other user’s healthier-looking fish. Of concern within the current study is how low levels of PA may have resulted in youth not wanting to display or interact with their 3D-printed models of PA as consequence of it negatively impacting their social identity, such as feelings of pressure and guilt for not achieving enough PA [20]. However, given that a low proportion of youth (*n* = 6) expressed frustration towards their inactivity displayed on the 3D-printed models in comparison to peers, the present findings may also be considered as an indication that children and adolescents are not internalizing messages relating to the health benefits of exercise.

In accord with previous studies investigating the barriers and facilitators to PA promotion [58,59], the current study highlights the important influential role of significant others in a participant’s PA levels. Interestingly, the 3D models were reported to increase significant other’s understanding of a participant’s PA levels relative to the guidelines, enabling peer and familial support and encouragement to increase PA levels, where necessary. This influence of significant others is likely to follow the principle of co-experience, whereby experiences are highlighted for shared attention, and become part of the social interpretation process that influences the meaning attributed to the experience by the individual and others [53]. Indeed, it has previously been reported that children are more likely to engage in more intense PA when in the company of peers or close friends [60]. Furthermore, systematic reviews have demonstrated strong relationships between the PA levels within a friendship group [61,62,63], supporting the concept of ingroup norms. Taken together, these findings highlight the importance of considering the role of significant others in the long-term efficacy and interpretation of 3D-printed models of PA.

### Strengths and Limitations

The focus groups employed within the current study provided an effective means by which to explore the relationship between identity and 3D models in children, through the facilitation of open discussion. The inclusion of both primary and secondary school students allowed for a more nuanced understanding of the relationship between identity and the 3D-printed models of PA. Nonetheless, certain limitations need to be taken into consideration when interpreting the current findings. Although the views of the primary school children were invaluable, it is important to note that the depth of discussion was less with this sub-group. A greater tendency to simply agree with others rather than elaborate with examples was observed. However, clarification and expansion of points raised was facilitated ‘in situ’, with the focus groups, to minimise any impact. Furthermore, the focus groups were conducted at the end of the intervention; richer data may have been derived if more regular discussions were held with participants to explore the influence of the 3D models on the development of personal and social identities. Regular discussions may have captured the influence of the 3D models on behaviour contemporaneously, especially through the use of in-group norms.

## 5. Conclusions

3D-printed models of PA can play an important role in the development of both personal and social identity through using autotopographical processes to facilitate social comparison and youth interactions with their parents and peers. Whilst the impact on long-term changes to youths PA identity are yet to be established, the use of 3D-printed models presents a novel concept and intervention approach that prompted reflection on children and adolescents’ own behaviour. Autotopographical approaches may be a vital element of successful behaviour change engagement strategies in these age groups.

## Data Availability

The data that support the findings of this study are available from the corresponding author upon reasonable request.

## Supplementary Materials

The following supporting information can be downloaded at: https://www.mdpi.com/article/10.3390/ijerph20021530/s1, File S1: 3D Printing Physical Activity Intervention: Focus Group Questions.
