# Supplementary material for: 3D-Printing Physical Activity in Youth: An Autotopographical Approach to Behaviour Change
Source: Int J Environ Res Public Health. 2023 Jan 14;20(2):1530. doi: 10.3390/ijerph20021530 (PMC9861837; doi:10.3390/ijerph20021530)
Supplement: Supplementary file 1 [file ijerph-20-01530-s001.zip › ijerph-2135341-supplementary.pdf]

3D Printing Physical Activity Intervention: Focus Group Questions

Ground Rules for Participants:

Year 3 Script:

“ So, I’m just quickly going to go over the rules of how this focus group discussion works. The first and most helpful rule is that only one person speaks at a time, but don’t worry, there’s no right or wrong answer to these questions and its quite likely you’ll all have different answers anyway! But, if you have something you would like to add to another person’s answer, please wait until they have finished and then speak.

When speaking, please make sure you don’t cover your mouths or have your fingers up your nose, otherwise I won’t be able to understand what you’ve said (jokingly demonstrating)! If you don’t have an answer, it’s ok, just tell me that you’re not sure or you don’t know. When one of you is speaking make sure to listen and not to interrupt them, as you will all get a turn to give your answer.

…Most importantly, remember to relax and enjoy yourself, it’s all about telling me what you think and how you see things, just like having a discussion as you would do in class. The reason I’m filming this group discussion is so that I can go back over what you guys say because I’ve got a really bad memory and won’t remember all the important things you’ve told me! Only myself and other members of the team will be able to see them!”

Year 9 Script:

“ So, now I’m just going to go over some of the rules of the focus group interview. It is very important that you speak one at a time and that you listen carefully, but remember there’s no right or wrong answers to these questions, it’s all about what you think! Don’t worry though if you don’t have an answer to one of the questions, just tell me your unsure or you don’t know. However, if you have something you would like to add to someone else’s answer please wait until they have finished and then put your hand up and I will come to you. Please make sure you’re not cover your mouth when speaking, otherwise I will have a really hard time understanding what you’ve said (said light-heartedly).

…Most importantly, I want you guys to feel relaxed and confident to speak out, just imagine it’s a discussion, just as you would do in class. I will be recording everything and this is because its very unlikely that I will be able to remember all the important things you’ve said to me (said jokingly). Only myself and members of the research team will be allowed to see the recordings”

- Start of Questions -

Icebreaker:

"Before I get started with the questions, if I can get each one of you to introduce yourselves to the camera by giving your name and something you like to do outside of school, like your favourite hobby or even a silly fact about yourself? I will get things started, so, my name is Sam and I when I was younger I used to dress up as Batman and run around in circles singing the Batman theme tune, dun-nuh-dun-nuh-dun-nuh-dun-nuh-batman! Ok, would you like to go next? (nod at the first participant, to the left).

Brilliant, Ok I will start with the questions now, so…"

1. What was the one thing you enjoyed the most about the taking part in the study? Why?

2. What was the one thing you enjoyed the least about taking part in the study? Why?

3. What was your first thing you thought/reaction to the 3D printed model when you saw it? (Prompts: Why did you think that? Was it what you imagined?)

4. How does being physically active make you feel about your body?

5. How does being physically active make you feel in your mood? (Prompt: do you feel more sad, more happy?)

Now, think back to before you took part in the study…

6. What did you think physical activity was before you took part in the study?

7. What do you think physical activity is now? (Prompt: after the study? Why do you think your thoughts have changed/not changed?)

8. Can you describe to me what happens to your body when you do vigorous/hard levels of physical activity?

9. Can you describe to me what happens to your body when you do moderate/medium levels of physical activity?

10. Do you think you have a better understanding of how much physical activity you should be achieving every day after completing the study? Why is that?

11. How, if at all, have the 3D printed models made you think about other ways of being active, besides sports?

12. Did you take up any new physical activities during the time you spent taking part in the study? Why?

13. Do you think you are now more motivated to be physically active than before you took part in the study? Why?

14. Did you compare your own models? (Prompt: What about with friends and classmates? How did you feel when you compared your 3D models with friends/classmates?)

15. What did you parents/carers say about your 3D models? (Prompt: did they like them?)

16. What did you do with all your 3D printed models? (Prompt: in your room? Hang them up?)

17. What do you think about the design of the 3D model? What, if anything, would you change about it? How?

18. What would you change, if anything, about the project? (Prompt: Would you like more interaction/hands on with the 3D printer and the process? Why?)

<u>TASK 1</u>: <u>Model Recall</u>

Right, we are almost at the end of the discussion, I just have one small task for you all to complete followed by three more questions!

So what I want you to do here is to label the diagram of the Sun model/Bar chart model by drawing arrows to show what you think each part represented. I am then going to ask each one of you to describe what you think the parts represent and don't copy each other!

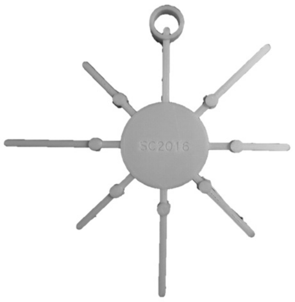

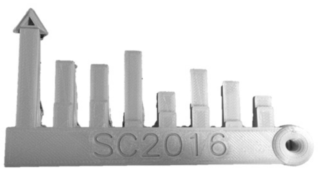

19. Is there anything you don't understand about the models? (Prompt: What do you find the hardest part to understand on the model?)

20. Is there anything you would like to ask me about the study?

- End of Questions -
